# EHMTI-0230. Characteristics of menstrual and nonmenstrual migraine attacks in women with menstrual migraine

**DOI:** 10.1186/1129-2377-15-S1-D67

**Published:** 2014-09-18

**Authors:** KG Vetvik, EA MacGregor, J Šaltyté Benth, AC Lundqvist, MB Russell

**Affiliations:** 1 Head and Neck Research Group Research Centre and Institute of Clinical Medicine, University of Oslo and Akershus University Hospital, Lorenskog, Norway; 2 Centre for Neuroscience and Trauma Blizard Institute, Barts and the London School of Medicine and Dentistry, London, UK; 3 Institute of Clinical Medicine and HØKH Research Centre, University of Oslo and Akershus University Hospital, Lørenskog, Norway; 4 Institute of Clinical Medicine and Head and Neck Research Group and HØKHResearch Centre and Department of Neurology, University of Oslo and Akershus University Hospital, Lørenskog, Norway; 5 Institute of Clinical Medicine and Head and Neck Research Group Research Centre, University of Oslo and Akershus University Hospital, Lørenskog, Norway

## Introduction

The International Classification of Headache Disorders 3β defines menstrual migraine (MM) as attacks of migraine without aura occurring on day 1±2 of the menstrual cycle in ≥2/3 menstruations. Previous studies show conflicting results regarding possible differences between menstrual and nonmenstrual migraine attacks.

## Aim

To compare characteristics of menstrual and nonmenstrual migraine attacks in women prospectively diagnosed with MM.

## Method

237 women from the general population with self-reported migraine in ≥50% of their menstruations were interviewed and diagnosed by a neurologist. Subsequently they were asked to complete a three month prospective headache- and menstruation diary. When a headache occurred, the women recorded information about pain intensity, quality and location, time of onset and end of headache, associated symptoms, aggravation by routine physical activity, medical treatment and sick leave. In addition, they were asked to record each day of uterine bleeding.

## Results

Of the 123 (52%) returned diaries, 42 were excluded due to oligoamenorrhoea (n=36), no migraine (n=4) or incompleteness (n=2). Among the remaining 81 women, 56 fulfilled the diagnostic criteria for MM. In these 56 women, menstrual migraine attacks were more painful on a 0-10 scale (coefficient 0.34, 95% CI 0.02-0.65), longer lasting (coefficient 10.91 hours, 95% CI 5.68-16.14), and required more doses of symptomatic treatment (coefficient 1.09 doses, 95% CI 0.50-1.68) compared to nonmenstrual attacks. Additionally, they were more often associated with nausea (OR 2.22, 95% CI 1.35-3.65).

## Conclusion

In women with MM, menstrual migraine attacks are more severe than nonmenstrual attacks.

No conflict of interest.

